# The contributions of Nettie Stevens to the field of sex chromosome biology

**DOI:** 10.1098/rstb.2021.0215

**Published:** 2022-05-09

**Authors:** Sarah B. Carey, Laramie Aközbek, Alex Harkess

**Affiliations:** ^1^ Department of Crop, Soil, and Environmental Sciences, Auburn University, Auburn, AL 36849, USA; ^2^ HudsonAlpha Institute for Biotechnology, Huntsville, AL 35806, USA

**Keywords:** sex chromosomes, dioecy, cytology, genomics

## Abstract

The early 1900s delivered many foundational discoveries in genetics, including re-discovery of Mendel's research and the chromosomal theory of inheritance. Following these insights, many focused their research on whether the development of separate sexes had a chromosomal basis or if instead it was caused by environmental factors. It is Dr Nettie M. Stevens' *Studies in spermatogenesis* (1905) that provided the unequivocal evidence that the inheritance of the Y chromosome initiated male development in mealworms. This result established that sex is indeed a Mendelian trait with a genetic basis and that the sex chromosomes play a critical role. In Part II of *Studies in spermatogenesis* (1906), an XY pair was identified in dozens of additional species, further validating the function of sex chromosomes. Since this formative work, a wealth of studies in animals and plants have examined the genetic basis of sex. The goal of this review is to shine a light again on Stevens’ *Studies in spermatogenesis* and the lasting impact of this work. We additionally focus on key findings in plant systems over the last century and open questions that are best answered, as in Stevens' work, by synthesizing across many systems.

This article is part of the theme issue ‘Sex determination and sex chromosome evolution in land plants’.

## 1. Introduction

For over a century, uncovering the genetic basis for the development of the separate sexes has been a lively area of research. How a single species develops two strikingly different forms captivated early naturalists, like Carl Linnaeus and Charles Darwin, but it was not until the early 1900s that sex was shown to have a genetic basis. The pivotal study that provided this evidence was *Studies in spermatogenesis* (1905) by Dr Nettie M. Stevens [1]. In this two-part piece, Stevens showed, through careful cytological examination, that the inheritance of the Y chromosome is correlated with male development in dozens of insect species. Despite the importance of this work, and over 6000 peer-reviewed articles on the topic of sex chromosomes since (Web of Science, accessed 20 August 2021), *Studies in spermatogenesis* has been cited fewer than 100 times (Google Scholar, accessed 24 July 2021). Here, we aim to reilluminate interest in this eloquent body of work and the decisive importance of Stevens' research to the topic of sex chromosomes. We next discuss the outpouring of studies on sex chromosomes in diverse plant systems after the publication of *Studies in spermatogenesis*, and posit that the future of studying sex chromosomes should follow the lessons of past and current researchers by examining many independent evolutions across kingdoms.

## 2. Nettie Stevens’ career

**Figure 1.**
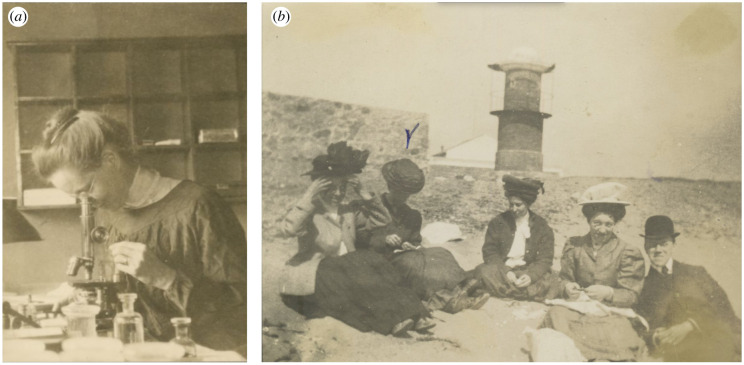
Photographs of Dr Nettie M. Stevens. (*a*) Stevens looking through her iconic microscope (1909), Bryn Mawr College Special Collections, PA_Stevens_Nettie_005. (*b*) Alice Boring, Nettie Stevens and colleagues at a beach near Capo di Messina (1909), Bryn Mawr College Special Collections, PA_Stevens_Nettie_001.

Nettie Maria Stevens was born on 7 July 1861 in Cavendish, Vermont, USA (figure 1). Stevens started her education at Westford Academy (1872–1880) and Westfield State Normal School (now Westfield State University; 1881–1883), to prepare for a career in teaching, and for the next decade or so Stevens worked as a teacher or librarian [2]. She saved enough money to continue her education, and in 1896 she began at Stanford University (then called Leland Stanford Jr University), earning both Bachelor and Master degrees (1896–1900). It was during this time at Stanford that Stevens' cytological and histological research took off while spending her summers working at the Hopkins Marine Station. In 1901, she published her first manuscript histologically describing ciliates, where through her detailed observations across the life cycle, she identified two new species [3].

The turn of the twentieth century was a transformative time for cytogenetic studies. Gregor Mendel's foundational research on heredity in pea plants, establishing the laws of segregation and independent assortment in reproductive cells [4], had recently been rediscovered by Carl Correns, Erich von Tschermak and Hugo de Vries [5–7]. Only a few years later Walter S. Sutton and Theodor Boveri independently showed that the behaviour of chromosomes during meiosis could be the basis for such Mendelian inheritance [8,9]. Though not all biologists were sold on the role of chromosomes in heredity based on these works alone, Stevens was quick to adopt these findings into her research.

Nettie Stevens continued her education at Bryn Mawr (Pennsylvania, USA), which by many accounts was an ideal place for biological research. Bryn Mawr was a relatively new school at this time, established in 1885 as one of the Seven Sister Schools, but had employed two well-known biologists in succession: Edmund Beecher Wilson, who would later author the acclaimed *The cell in development and inheritance* (1896) [10] and Thomas Hunt Morgan, future Nobel Laureate (1933) and ‘Father of Modern Genetics'. Though Wilson left for Columbia University before Stevens started, Morgan became Stevens' doctoral advisor, and the three collaborated closely. Soon after starting, in 1901, Stevens received the Bryn Mawr President's European Fellowship, which provided funding to research at Naples Zoological Station with Theodor Boveri, who at the time was working on his contributions to the chromosomal theory of inheritance. Stevens' doctoral thesis built on her Master's work, expanding to new species and varieties of ciliates, where she described microanatomy and regeneration [11]. In 1903, Nettie Stevens received her PhD.

Over the next several years, Stevens continued her upward trajectory and notability as a scientist. In 1903, Stevens applied for and received a grant to specifically study sex determination by chromosomes [12], the research published in *Studies in spermatogenesis*. In 1904, she became a postdoctoral research assistant with the Carnegie Institute of Washington and then returned to Bryn Mawr as a research associate. Her research continued to focus on cytological analyses throughout spermatogenesis, development and regeneration. Interestingly, Stevens may have also been one of the first scientists to discover B chromosomes, suggesting a possible relationship between these and sex chromosomes [13,14]. In 1905, her manuscript focusing on the germ cells of aphids won the Ellen Richards Prize given by the Association for Maintaining the American Woman's Table at the Zoological Station at Naples [15]. In 1910, Stevens was listed in the top 1000 ‘men of science’, being one of 18 women recognized that year [16]. By 1912, Stevens was finally offered a research professorship at Bryn Mawr, but before she began this new role, she died of breast cancer at the age of 50 (4 May 1912).

Without a doubt, despite her life and career tragically being cut short, Stevens made an extraordinary impact on the field of biology. In the 11 years between Stevens' first publication and her passing, she published at least 38 manuscripts [2]. Stevens’ contributions have not been completely lost to time. In 1994, Stevens was inducted into the National Women's Hall of Fame and in 2017 Westfield State University opened the Dr Nettie Maria Stevens Science and Innovation Center. Stevens was a remarkably accomplished scientist with many foundational discoveries, though her best-known is about the role of sex chromosomes.

### (a) Studies in spermatogenesis

The development of the sexes was an area of substantial interest by the end of the ninteenth century. As Wilson described it, ‘The phenomenon of sex is so nearly a universal one that it may be assumed to make some appeal to the interest of biologists in every field of inquiry’ [17, p. 53]. Many researchers began investigating the leading theories behind sex determination, principally whether there is a genetic underpinning or if external environmental factors are involved. While today there are some species for which a form of environmental sex determination has been identified [18,19], most species with gonochory or dioecy have a genetic basis.

The beeline that resulted in the identification of sex chromosomes started in 1891, when Hermann von Henking found in the firebug, *Pyrrochoris apterus*, that during meiosis half of the sperm inherited 11 chromosomes and the other half 12. Von Henking called this twelfth chromosome the ‘X-element’ [20]. Less than a decade later, in 1899, McClung proposed the term ‘accessory chromosome’ for this element [21] and in 1902 he presented a theoretical framework for the involvement of this sperm accessory chromosome in the sex of an organism,

A most significant fact, and one upon which almost all investigators are united in opinion, is that the element is apportioned to but one half of the spermatozoa. Assuming it to be true that the chromatin is the important part of the cell in the matter of heredity, then it follows that we have two kinds of spermatozoa that differ from each other in a vital matter. We expect, therefore, to find in the offspring two sorts of individuals in approximately equal numbers, under normal conditions, that exhibit marked differences in structure. A careful consideration will suggest that nothing but sexual characters thus divides the members of a species into two well-defined groups, and we are logically forced to the conclusion that the peculiar chromosome has some bearing upon this arrangement. [22]

Regarding the accessory chromosome, McClung (1902) also writes that ‘Its careful and uniform division during the mitoses of all the spermatogonia suggests anything but an unimportant structure’ [22]. Studies focusing on identifying accessory chromosomes in diverse systems swelled. Louise Wallace identified a double accessory chromosome system in the spider *Agalena naevia* [23]. Frederick Paulmier considered the accessory chromosome to be degrading and disappearing from a species because he observed that it fails to divide and is not equally represented in the final spermatocyte mitosis [24]. Likewise, Thomas Montgomery thought the accessory chromosomes ‘… are in the process of disappearance, in the evolution of a higher to a lower chromosomal number’ [25]. Discussion on whether the accessory chromosomes were involved in sex determination continued, but the direct evidence for its role had yet to be shown.

**Figure 2.**
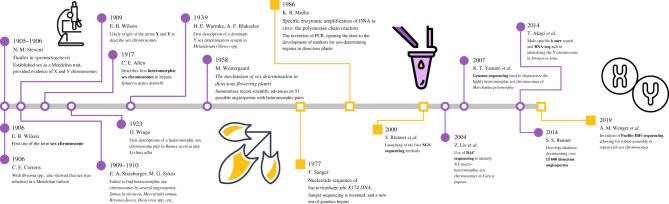
Key events for visualizing sex chromosome research in plants over time. Purple circles indicate empirical findings and yellow squares technological advances that have set the foundation for discovery in sex chromosome research. The timeline begins with Stevens' discovery of sex chromosomes, followed by the wave of cytological research that followed her, including the first descriptions of a heteromorphic sex chromosome pair in a liverwort (1917) and in angiosperms (1923). With the development of PCR and modern sequencing techniques, the identification of sex chromosomes diverged from traditional cytological techniques and moved toward marker-based as well as whole-genome approaches. This has led to a new renaissance of sex chromosome research not unlike the one Stevens began in 1905. An expanded timeline can be found in electronic supplementary material, table S1. NGS, next-generation sequencing.

*Studies in spermatogenesis* was published as a two-part book, with the first part released in 1905 (figure 2) [1]. Importantly, Stevens tracked the behaviour of the accessory chromosome across different orders of Coleoptera and deduced its inheritance pattern through cell division. Part I of *Studies in spermatogenesis* included chromosome squashes from termites (*Termopsis angusticollis*), sand crickets (*Stenopelmatus* spp.) and croton-bugs (*Blattella germanica*), and indeed, in *Stenopelmatus* and *B. germanica*, Stevens found evidence of the accessory chromosomes. But it is mealworms (*Tenebrio molitor*) that Stevens described as the most interesting group studied in her 1905 publication, for what she found differed from that of the accessory chromosomes described by McClung. She writes that ‘In both somatic and germ cells of the two sexes there is a difference not in the number of chromatin elements, but in the size of one, which is very small in the male and of the same size as the other 19 in the female’ [1]. Stevens reasons that,

Since the somatic cells of the female contain 20 large chromosomes while those of the male contain 19 large ones and 1 small one, this seems to be a clear case of sex-determination, not by an accessory chromosome, but by a definite difference in the character of the elements of one pair of chromosomes of the spermatocytes of the first order, the spermatozoa which contain the small chromosome determining the male sex, while those that contain 10 chromosomes of equal size determine the female sex. This result suggests that there may be in many cases some intrinsic difference affecting sex, in the character of the chromatin of one-half of the spermatozoa, though it may not usually be indicated by such an external difference in form or size of the chromosomes as in *Tenebrio*. [1]

One of the virtues of Nettie Stevens' work is the diversity of species where she observed the segregation of different sex chromosome systems. Stevens published part II of *Studies in spermatogenesis* in June of 1906, where she studied the spermatogenesis of 23 more species in Coleoptera, and in August 1906 a footnote was added containing results for 19 more [26]. In this second part, Stevens found that 86% of the species studied are characterized by having heterochromosomes and the remaining had accessory chromosomes in male germ cells [26]. On the accessory chromosomes (referred to here as ‘odd chromosome’) Stevens writes,

The odd chromosome, so far as it has been studied, behaves precisely like the larger member of the unequal pair without its smaller mate. In the growth stage it remains condensed and either spherical or sometimes flattened against the nuclear membrane. In the first maturation mitosis it is attached to one pole of the spindle, does not divide, but goes to one of the two second spermatocytes. In the second spermatocyte it divides with the other chromosomes, giving two equal classes of spermatids differing by the presence or absence of this odd chromosome. [26]

In this section, Nettie Stevens uses the term ‘mitosis' to describe what is now known as meiosis I and II in the spermatocytogenesis, where primary spermatocytes (2*n*) divide into secondary spermatocytes in meiosis I and spermatids in meiosis II. Interestingly, the term ‘meiosis’ (from the Greek *με*ί*ωσι*ς, ‘lessening’) was not coined until 1905 by cytologists John Farmer and John Moore, explaining the absence of this term in Stevens' analysis [27]. She demonstrates that these divisions lead to the ‘odd chromosome,’ labelled as ‘*x’* on her plates, segregating according to Mendelian principles in meiosis I and II. Likewise, this Mendelian behaviour was found for the pair of heterochromosomes that she labels as ‘*l*’ (for large) and ‘*s*’ (for small), which later became known as ‘X’ and ‘Y’ chromosomes.

The implications of her observations and deductions are elegant and profound: these odd chromosomes (*x*) or heterochromosomes (*l* or *s*) follow Mendel's laws of inheritance, and the presence (or absence) of these chromosomes corresponds to sex determination. The logic behind this is eloquently noted in her discussion, when she states that *‘*It is therefore evident that an egg fertilized by a spermatozoon (1) containing the small member of an unequal pair or (2) lacking one chromosome, must develop into a male, while an egg fertilized by a spermatozoon containing the larger element of an unequal pair of heterochromosomes or the odd chromosomes must produce a female’ [26]. In 1905, the same year as part I of *Studies in spermatogenesis* was published, E. B. Wilson also published a study on the sex chromosomes in Hemiptera [28]. In his piece, Wilson also showed that males possess an unequal pair of chromosomes, the smaller of which he called the ‘idiochromosomes'. Wilson added a footnote to his 1905 piece acknowledging Stevens' findings.

The discovery, referred to in a preceding footnote, that the spermatogonial number of Anasa is 21 instead of 22, again goes far to set aside the difficulties [of McClung's hypothesis] here urged. Since this paper was sent to press I have also learned that Dr N. M. Stevens (by whose kind permission I am able to refer to her results) has independently discovered in a beetle, Tenebrio, a pair of unequal chromosomes that are somewhat similar to the idiochromosomes in Hemiptera and undergo a corresponding distribution to the spermatozoa. She was able to determine, further, the significant fact that the small chromosome is present in the somatic cells of the male only, while in those of the female it is represented by a larger chromosome. These very interesting discoveries, now in course of publication, afford, I think, a strong support to the suggestion made above; and when considered in connection with the comparison I have drawn between the idiochromosomes and the accessory show that McClung's hypothesis may, in the end, prove to be well founded. [28, p. 403]

While Wilson's research was published a few months before Stevens', some give Stevens the credit for the discovery of sex chromosomes because her conclusions were firmer [12]. Regardless of whether the discovery for the role of sex chromosomes should be shared between Stevens and Wilson, as the two independently arrived at these results in 1905, Stevens was certainly the first to concretely show that the Y chromosome was involved in sex determination. Her work provided a molecular and cytological framework for supporting the earlier hypothesis put forth by Carl Correns after crossing the dioecious vine *Bryonia* that sex was, indeed, a Mendelian trait [29]. McClung had incorrectly asserted that the accessory chromosome was a male determiner [22]. Wilson maintained environmental roles [29]. In Stevens’ own words,

Wilson suggests as alternatives to the chromosome sex according to Mendel's Law, (1) that the heterochromosomes may merely transmit sex characters, sex being determined by protoplasmic conditions external to the chromosomes; (2) That the heterochromosomes may be sex-determining factors only by virtue of difference in activity or amount of chromatin, the female sex chromosome in the male being less active. [26]

Over the next several years, more studies in spermatogenesis were undertaken by Stevens and her colleagues. Stevens was the first to identify the heterochromosomes of *Drosophila melanogaster* (then called *Drosophila ampelophila*) and other flies [30,31]. Even more heteromorphic pairs were found in earwigs (*Forficula auricularia*) [32] and guinea pigs [33]. Stevens' rigorousness and tenacity to uncover the role of sex chromosomes were apparent, and her depth of knowledge of the field unmatched. Upon learning about lagging chromosomes, Stevens carefully re-examined aphids, revealing the lagging member was in fact a heterochromosome, revoking her previous findings that these species lacked evidence for any [34]. But, not in all species could heterochromosomes be identified; such was the case in mosquitoes [35]. At Bryn Mawr, Stevens advised doctoral student Alice M. Boring (figure 1), who notes in her dissertation that while at Woods Hole in 1905, Stevens suggested Boring study the spermatogenesis of many more species of insects [36]. Indeed, Boring's PhD research focused on the spermatogenesis of 22 species, finding that all had the ‘odd chromosomes' [36]. Later Boring would study chicken spermatogenesis, where a clear pair of neither heterochromosomes nor accessory chromosomes could be identified [37]. Over a decade after Stevens' death, Boring found Stevens’ notes on her independent examinations of chicken [38]. As it turns out, the lack of heterochromosomes found in chicken spermatogenesis is because they have a ZW system, which was shown by Michael F. Guyer's studies in oogenesis in 1916 [39].

Across these foundational research pieces and more, many different terms were used to describe what we now refer to as sex chromosomes, an issue raised by researchers of the time. ‘Since the discovery of peculiarly modified chromosomes in certain of the insects a great variety of names has been proposed for them, and most of these suffer from a quite unnecessary length. My own earlier terms "heterochromosome" and "chromatin nucleolus" were cumbersome, and "accessory chromosome" and "heterotropic chromosome" sin equally in this regard, while "special chromosome" and "idiochromosome" are no way self-explanatory.’ [40]. In 1906 Wilson first used the term ‘sex chromosome’ [41] and by 1909 used ‘X’ and ‘Y’ to delineate between the heteromorphic pair [17]. Confusion about the term ‘sex chromosome’ and what it represents continues today (for a discussion on definitions, see [42]). By casting such a wide net of species diversity, scientists from each of these independent and complementary studies had stumbled upon the foundation of the modern diversity of sex chromosome systems, including XX/XO (dosage) systems where chromosome number changes between males and females, XX/XY systems where the heterogametic sex chromosome pair is found during spermatogenesis in males, and ZZ/ZW systems where the heterogametic pair is found in females during oogenesis.

## 3. The prismatic sex chromosomes of plants

**Figure 3.**
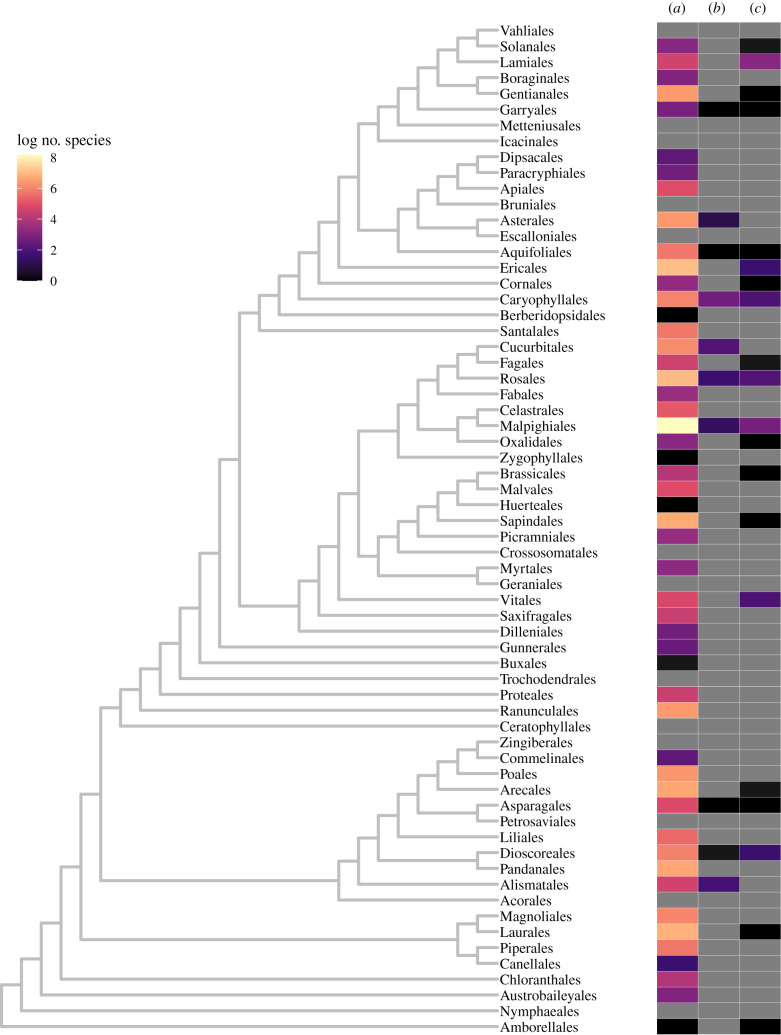
Dioecious angiosperm orders studied to date. The heatmap shows the number of species in log scale and is mapped onto the topology from Angiosperm Phylogeny Group IV [45] using ggtree v. 3.0.4 [46]. (*a*) dioecious species within each order [44], (*b*) species with heteromorphic sex chromosomes identified through cytological approaches, (*c*) dioecious species with at least one genome reference in the NCBI Assembly database (accessed 30 August 2021).

Undoubtedly, Nettie Stevens' research transformed animal genetics. The ground-breaking impact it had on plant genetics, however, is equally significant even if less obvious. Shortly after *Studies in spermatogenesis* was published, studies focusing on potential sex chromosome systems in plants burgeoned. Unlike animals, separate sexes, or dioecy, is rarer in angiosperms, occurring in approximately 6% of species with 34 clades—such as *Diospyros* (700 species) and *Pandanus* (600 species)—and sometimes entire families (e.g. Myristicaceae), accounting for roughly 43% of the dioecious angiosperms [43]. Even with its uncommonness, dioecy has evolved hundreds of independent times in angiosperms, while in other plant lineages, such as those of the bryophytes and gymnosperms, it appears to be more common [44] (figure 3). Despite being seemingly disadvantageous for a sessile organism, dioecy is still the dominant reproductive strategy for critical fruit (fig), nut (pistachio), vegetable (asparagus), ornamental (gingko) and special products crops (hops, hemp) among many other species valuable to forestry, conservation, and bioremediation efforts [47]. While botanists, farmers and horticulturalists had always been acutely aware of this trait, Stevens’ work was the first to provide a foundation for exploring its genetic basis in plants.

A rush of cytological studies emerged as botanists re-examined the karyotypes of dioecious species (electronic supplementary material, table S1). Some of the earliest records of this frenzy come from 1909, when Eduard Strasburger and Mary G. Sykes observed the absence of heteromorphic sex chromosomes in *Mercurialis annua, Bryonia dioica* and *Spinacia oleracea* [48–50]. It took until 1917, more than decade after Stevens' discovery of sex chromosomes, for Charles E. Allen to confirm the presence of heteromorphic sex chromosomes in the liverwort *Sphaerocarpos* [51]. A slew of cytological studies followed (figure 3), suggesting the presence of heteromorphic sex chromosomes in 68 plants and their absence in 46 plants by 1940 [52]. In 1958, Mogens Westergaard proposed a standard to temper the continuous outpouring of scantly supported claims of heteromorphic sex chromosome pairs. He argued that observations of such pairs are only valid if the heteromorphic pair is observed in the meiotic cycle of the heterogametic sex and not the homogametic sex, and if the sex chromosomes are also observable in the somatic cells of both sexes [53], as was done in Stevens’ *Studies in spermatogenesis* [1]. Since this time, only 19 species have been confirmed to have heteromorphic sex chromosomes, such as *Cannabis*, *Humulus*, *Silene*, *Trichosanthes* and *Rumex*. Species are being added and removed from this list as the meaning of ‘heteromorphic’ continues to evolve in genomic literature [42]. In the decades following Westergaard's review, the diversity of species studied on sex chromosomes in plants decreased as *Silene*, *Spinacia* and *Asparagus* spp. emerged as model systems.

Sex chromosome research has always been limited by the capabilities of microscopic or genomic technologies. The absence of heteromorphic sex chromosomes in many dioecious species presented a distinctive challenge that would not be taken on until the advent of modern sequencing techniques in the early 2000s (figure 2). Yet, the cytologists of Stevens' day did not lack an abundance of surprising and often bewildering observations of heteromorphic sex chromosomes. As with many animals, plants also exhibit a wide variety of karyotypes that do not follow the standard XY or ZW systems. Hitoshi Kihara and Tomowo Ono first described the XX/XY_1_Y_2_ system in *Rumex acetosa* in 1925 [54]. Soon after, Öjvind Winge elaborated on the polytypic qualities of *Humulus* species, which are well-known for their markedly variable cytotypes [55]. Unusual systems continued to intrigue researchers into the late 1900s, such as the sex-associated floating translocation complexes in *Viscum*, where four, six and sometimes eight chromosomes form multivalent rings at meiosis [56]. The UV systems are similarly variable, with many homomorphic and heteromorphic pairs found by C. E. Allen [57], as well as multiple systems like the U_1_U_2_/V found in *Frullania dilatata* [58,59]. Methods for the visualization of sex chromosomes have dramatically improved since the days of Stevens’ Carl Zeiss Jena 8261 compound monocular microscope (figure 1; Bryn Mawr College Special Collections). Today, modern technologies, such as PacBio HiFi sequencing, have opened the door to more robust assembly of repeat-rich sex chromosomes and made research on homomorphic sex chromosomes, sex-determining regions (SDRs) and pseudo-autosomal regions more accessible than ever before [60].

In the last several decades, genomic approaches have shed light on many previously unexamined or unidentified plant sex chromosome pairs (figures 2 and 3). The first plant genome reference for the hermaphroditic species *Arabidopsis thaliana* was published in 2000 [61], and quickly several sex chromosome assemblies followed, including for papaya and the common liverwort [62,63], with whole-genome references eventually to follow [64,65]. Today over 100 dioecious angiosperm genome references, at various levels of contiguity, are available on NCBI (figure 3). Yet, only a fraction of these references have been used to examine the sex chromosomes.

Genomic analyses of plant sex chromosomes have addressed many theories developed for this kingdom. Because of the thousands of independent origins of plant sex chromosomes and few heteromorphic pairs identified cytologically (figure 3), the age of most evolutions was thought to be recent. The expectation is that heteromorphic pairs have had sufficient time for degeneration, or gene loss, to have occurred on the sex-specific chromosome to suggest older origins [66]. Such is the case in the older, cytologically heteromorphic pairs of *Humulus lupulus* [67], *Phoenix dactylifera* [68] and *Silene* *latifolia* [69]. In some cases, given enough time, the Y (or W) can be completely lost, transitioning to the XO (or ZO) system seen in studies of the ‘accessory chromosomes' [42]. Though, curiously, to our knowledge, no plant species has been reliably identified as having a dosage (e.g. XO) system. Instead, homomorphic sex chromosomes are expected to have more recent origins, with little to no gene loss on the Y (or W). Consistent with this, many species studied have recent origins of sex chromosomes, within the last 5 million years, such as in *Asparagus officinalis* [70]*, M. annua* [71] and *S. oleracea* [72], and so few (critical) genes have been lost from the Y that individuals with a YY karyotype remain viable [73–76]. However, some plant sex chromosomes defy these expectations. The moss UV sex chromosomes evolved hundreds of millions of years ago but are homomorphic in *Ceratodon purpureus* [77] and in *Cannabis sativa* the sex chromosomes share an origin with *H. lupulus* [67]; however, they are instead homomorphic.

The size of the non-recombining region also does not correlate with age in species studied to date [43], which may relate to haploid gene expression. Unlike animals, the plant life cycle consists of two separate generations, one haploid and the other diploid (i.e. alternation of generations), which has consequences for sex-specific development and sex chromosome evolution [76]. Because plants express genes in pollen or other haploid gametophyte stages, the non-recombining region of the sex chromosomes is expected to degenerate slower than is seen in animals [78,79]. Indeed, Mank suggests haploid expression in plants may represent the biggest difference known for sex chromosomes between these two kingdoms [80]. The *S. latifolia* sex chromosomes evolved over 10 million years, and while some genes have been lost on the SDR, the rate of loss is 60% lower than that of animals with a similar time since suppressed recombination occurred [81,82]. Estimates of divergence between XY genes in *Rumex* *hastatulus* suggest a minimum age of 9 million years [83], and while some genes have also been lost on the Y [84], pollen-expressed genes are significantly less likely to be lost than those expressed in diploid tissues [83,85]. The haploid *C. purpureus* UV sex chromosomes contain over 3400 genes each, half of which were shown to be expressed in the gametophytes [77]. In addition to haploid gene expression, the lack of degeneration could be due to the small size of the SDR seen in many plants, as degeneration is predicted to be faster when many genes are under selection [86,87].

Plant sex chromosomes are not without consequences from suppressed recombination. A consistent pattern found is an enrichment of transposable elements (TEs) and other repeats [77,88–90], which often accumulate in regions of low recombination [91]. In fact, in several species TE expansions have instead driven the Y chromosome to be larger than the X, such as in *Coccinia grandis* [92] and *S. latifolia* [93]. This pattern is counter to the smaller-Y heteromorphy found in the insects studied in Stevens' day. While many Y chromosomes in animals are also riddled with repeats, in well-studied species most of the genes have been lost [94–97]. Moving beyond analyses of single species, comparisons between sister species with a shared evolution of sex chromosomes, such as in *Coccinia* [98], will provide greater insight into these degenerative processes.

The genes underlying the transition to dioecy, and subsequently the evolution of sex chromosomes, are also an area of interest in plants. Given many dioecious species are economically important, or closely related to ones that are, uncovering the genes that control reproductive structures is useful to breeding programmes. Additionally, these genes amass critical insight into how sex chromosomes evolve. In theory, the transition from hermaphroditic flowers to dioecy can occur through two mutations: one affecting female fertility, or carpel development, and another affecting male fertility, or stamen development [53,99]. Recent evidence in several plant species supports this two-gene model, such as in *Actinidia deliciosa* [100], *A. officinalis* [101] and *P. dactylifera* [102]. Contrastingly, a few systems have strong evidence of a single gene initiating female versus male development, as shown in persimmons and poplars [103,104]. Complementary to the many independent evolutions of dioecy, in each of these species examined, different genes have been identified as sex-determining and they function at varying parts of floral development (see reviews in [43,105]). Studies of additional independent origins of sex chromosomes in plants may indeed identify more novel genes involved in carpel and stamen pathways. Undisputedly, there is a veritable array of sex chromosomes found in plants (figure 3 and electronic supplementary material, table S1) and every species examined garners new insight on these fascinating parts of the genome.

## 4. The future of sex chromosome studies is through a multi-kingdom lens

Across the species Nettie Stevens studied, she found many that contained what she expected to find after their first discovery in mealworms: a heteromorphic XY pair. As we can see in the plants described, many also fitted the theoretical mould, but there are always exceptions that make us question the ‘rules’ at play for sex chromosomes [106]. In Stevens' 1911 manuscript she writes, ‘At present, the all-important questions seem to me to be: What is the meaning of the differentiation of heterochromosomes in one form and not in others closely related? What has been the history of such differentiation where we have an unpaired heterochromosome or an unequal pair of heterochromosomes?’ She adds ‘… But in no case are we able to say when or how or why certain spermatogonial chromosomes became specially differentiated as heterochromosomes.’ [35].

Today these questions remain at the heart of most studies on sex chromosomes. What drives gene gain and loss from the SDR, and what is the tempo at which these processes tick? The insights from plant sex chromosomes have highlighted differences that exist between them and animal systems, though there are ample similarities [80]. Yet, there are many independent evolutions across plants from which we can uncover more. Future studies could focus on the many existing genome references where the sex chromosomes have not been closely studied (figure 3). Nearly half of the dioecious orders do not have even a single dioecious genome reference, let alone ones at the genus or species level, highlighting the need for more genomic efforts focused on dioecious species (figure 3). Attention on more animal species is just as pressing, as well the other kingdoms that we have not focused on here, such as protists and fungi. Most critically, to answer these ongoing questions that have been posed for nearly as long as sex chromosomes have been known, we need to take a note from Stevens' brilliant career and examine many isolates and many species across kingdoms.

There appears to be so little uniformity as to the presence of the heterochromosomes, even in insects, and in their behavior when present, that further discussion of their probable function must be deferred until the spermatogenesis of many more forms has been carefully worked out. [1]

## Data Availability

The R script and materials to generate figure 3 can be found at https://github.com/sarahcarey/angiosperm_dioecy.
